# Annotations

**Published:** 1898-12-03

**Authors:** 


### ANNOTATIONS.

### The Protection of Convalescents from Small-pox.

THE Grange Convalescent Hospital at Torquay has made a move in the right direction with the object of protecting its inmates from infection by small-pox. The institution has for long enforced the ordinary rule that no patient can be admitted without a medical certificate stating, among other things, that "he has not been suffering from, or recently been in contact with, infectious disease." As a still further protection, however, it has been decided that in future "no patient will be admitted unless he bears proof of satisfactory vaccination," and, as a notification of this rule, the above words are printed in red ink on every form of medical certificate. There is no doubt that it is a matter of extreme importance that those who are responsible for the care of convalescents, or even of those who are merely in a weak state of health, should exercise the greatest possible care to protect them from all forms of infectious disease. Not only are such patients peculiarly susceptible to infection, but, what is more important, they are in a condition which makes the occurrence of a febrile ailment a very serious matter to them. Hence the wisdom of the new rule—one which, we think, might well be made of wide application. The assembled wisdom of the nation has decided that the number of those who are susceptible to the most fatal and loathsome forms of small-pox shall be largely increased, and that the protection against the disease which can be given by universal vaccination shall be done away with. It now, therefore, becomes a matter of each for himself. Perhaps before long we shall find that mistresses will refuse to engage unvaccinated servants; that employers will decline to expose their works to the chance of stoppage by outbreaks of small-pox, and will engage none but vaccinated workmen; perhaps even the British workman, whose gullibility has been largely at the bottom of the present *impassé*, will some time or another learn to demand extra wages for working in a shop in which vaccination is not enforced. In the meantime those who undertake the responsibility of drawing together numbers of weakly folk are morally bound to do all they can to protect them from further attacks of sickness, and among other means to take care that they are not exposed to the risk of mixing with unvaccinated people.

### The Open Air Treatment of Consumption.

WITH the coming of winter, and with wind and rain and snow swirling all around us, come voices of protest against the attempt to undertake the outdoor treatment of consumption in England. One gentleman, writing to a contemporary, points out what the open-air treatment really involves, and urges that almost every necessary condition is wanting in England. The sun, he says, is often absent and without power; the air is often far from pure, and during the greater part of the winter is saturated with moisture; there is constant wind from all directions—south-west laden with moisture, north-east biting and often damp; and there are but few days in the winter and early spring in England when a patient with tuberculous lung disease could lie out of doors all day or walk about. Face which way the balconies or promenades in England will, they would be liable to be invaded with drifting rain or sleet, and blown out with gales of wind. This is very sad, and all the more so in that, so far as it deals with the English climate, it is very true. But that is not exactly the point. What we have to consider is not the badness of the English climate, but the very important fact—for we think that by now we may fairly call it a fact—that notwithstanding its vagaries many patients do better in the open than in the house. With this before us it is no use wringing our hands about the climate; what we have got to do is to circumvent it, and to build some structure in which patients may obtain as much good and as little evil from our wintry breezes as may be possible. Physicians and engineers should put their heads together and see what they can produce.

### Ill-behaved Patients.

THE evidence given at an inquest held last week at the Whitechapel Infirmary, on a man who died soon after being turned out of the London Hospital, draws attention to what is in fact a great difficulty in hospital management, namely, to the question what to do when a patient persists in disgusting and indecent behaviour. If a patient is in such a condition that he can safely be turned out, there can be no doubt as to the propriety of his being at once ejected. A hospital is for the treatment of the sick, and it is the duty of the managers to put a stop to anything that interferes with the peace and tranquillity necessary for their recovery. In doing this their action may sometimes appear harsh, but the good of the greatest number has to be considered, and, for this reason, patients who persistently misbehave evidently must be removed from the wards. Whether they must be turned into the street must depend on their condition. In the case we have referred to the patient was turned out of the London Hospital on Saturday, November 5th, for gross indecency; but he was so ill that he died in the Whitechapel Infirmary on the Monday following, and we cannot but feel that it was a grave error of judgment merely to put a man out into the street when he was so ill as this man must have been. In all large hospitals it must occasionally happen that foul-mouthed and obscene people are admitted, and although we do not suggest for a moment that such people should be retained longer than is necessary to make arrangements for their proper removal, we do think that a hospital should have some better means of getting rid of unwelcome patients than merely setting them down on the pavement and leaving them for the police to deal with. Besides showing the lack of proper means of isolation, this case also tends to emphasise what is a want in most of our hospitals, namely, a proper contingent of trained male attendants to take charge of various cases which cannot with propriety be handed over to the care of nurses and probationers. Nurses are brave and willing, and we know are ready without grumbling to put up with much, but there is a limit to what women should be asked to do, and the nursing of a foul-mouthed ruffian, even though his lewdness be but delirium, in a ward full of men is beyond the line. It seems fair to believe that if the London Hospital had possessed proper conveniences for isolation, and had had at its disposal a staff of male attendants, it would have been spared the censure of the jury in this case.

